# Physicochemical Characteristics of Citrus Seed Oils from Kerman, Iran

**DOI:** 10.1155/2014/174954

**Published:** 2014-07-17

**Authors:** Mohammad Reazai, Issa Mohammadpourfard, Shahrokh Nazmara, Mahdi Jahanbakhsh, Leila Shiri

**Affiliations:** ^1^Department of Food Safety and Hygiene, School of Public Health, Tehran University of Medical Sciences, Tehran, Iran; ^2^Department of Environmental Health Engineering, School of Public Health, Tehran University of Medical Sciences, Tehran, Iran

## Abstract

Recently, there has been a great deal of attention on usage, byproducts, and wastes of the food industry. There have been many studies on the properties of citrus seeds and extracted oil from citrus grown in Kerman, Iran. The rate of oil content of citrus seeds varies between 33.4% and 41.9%. Linoleic acid (33.2% to 36.3%) is the key fatty acid found in citrus seeds oil and oleic (24.8% to 29.3%) and palmitic acids (23.5% to 29.4%) are the next main fatty acids, respectively. There are also other acids found at trivial rates such as stearic, palmitoleic, and linolenic. With variation between 0.54 meg/kg and 0.77 mgq/kg in peroxide values of citrus seed oils, acidity value of the oil varies between 0.44% and 0.72%. The results of the study showed that citrus seeds under study (orange and sour lemon grown in Kerman province) and the extracted oil have the potential of being used as the source of edible oil.

## 1. Introduction

*Citrus* species and 1300 of other species, classified in 140 genera, are members of the family Rutaceae. The fruit is grown mainly in south of Iran which is characterized with warm and humid climate, which is perfect for growing citrus [1]. Currently only the juice of the fruit is commercially used and the seeds are considered as waste. In general, peels, seeds, and pulps (around 50% of the fruit) are dealt with as wastes, while, potentially, they can be source of valuable byproduct [2]. Around 85 million ton (MT) of different types of citrus are the annual production of the world and Iran's contribution to this volume is 650.000 MT (9% of global production) [3]. The species of the fruit are found to be of medical values and are also used in confectionary, toiletry, and perfume industry. Given the economic, medical, and dietary values of citrus seed oil, there has been a recent surge of studies on the chemical composition (fatty acid content in particular) of the oil of seeds of different species of* Citrus*. Many works have measured the oil content of citrus seeds: Tunisian citrus seeds (26.1–36.1%) [4], Brazilian Rangpur lime seeds (32.0–38.3%) [5], Egyptian citrus seeds (40.2–45.5%) [6], Tunisian sweet orange (51.8%) and lemon seeds (78.9%) [4], and Pakistani citrus seeds (27.0–36.5%) [7]. Taking into account the gravity of the subject under study and given the large number of similar studies in Iran, the present one measures the seed oils of more commonly found citrus fruits in Iran in an attempt to determine fatty acid composition of the neutral lipid classes of seed oils.

## 2. Material and Methods

Samples were collected from the citrus fruits grown in Kerman province, Iran, in September. The samples were sliced and the seeds were taken out, rinsed, and dried in oven (40°C, 24 hrs).

### 2.1. Reagents and Standards

Analytical grade chemical and solvent (Merck, Germany) were used in the study. In addition, pure standard FA methyl esters were obtained from Sigma Chemical Company.

### 2.2. Oil Extraction

About 100 gr of crushed and grounded seeds (powder) was put into Soxhlet extractor with 1 Li bottomed flask and a condenser. During 8 hrs of extraction, 0.5 Li of hexane and n-hexane was extracted at 40°C or less with a rotator vacuum evaporator [8].

### 2.3. Preparation of Fatty Acid Methyl Esters (FAMEs)

In preparation of methyl esters fatty acid form the seeds, AOCS official method was followed [9].

### 2.4. Fatty Acids Detection

By transforming fatty acids into FAMEs by GC (Unicam 4600, UK) attached with a flame ionization capillary column fused silica (30 m × 0.25 (I.D.) mm, film thickness: 0.22 *μ*m), FA composition was studied. As gas carrier, helium was used. Then the temperature of the sample was increased up to 180°C at 20°C/min and it remained at this temperature for 10 min and afterward, with the same rate, the temperature was increased to 210°C. Temperature of the detector and injector was set at 250°C [10].

### 2.5. Other Properties

Saponification value, acid value, iodine value, and peroxide value were determined according to AOAC 2005 [11].

## 3. Results 

Table 1 lists the composition of fatty acid and fat content of citrus seeds. As the results show, the oil content ranges between 40.3% and 41.9% for sour lemon and 33.4% and 34.2% for orange. The mostly found saturated component in all citrus seeds is palmitic acid. Its content varies for different types of seeds and on average it ranges between 26.9% in sour lemon seed and 27.13% in organ seed. In addition, the content of oleic acid of sour lemon was found between 24.8% (samples from Jiroft) and 28.5% (samples from Anbarabad) and in the case of orange it was found between 27.1% (samples from Qaleh Ganj) and 29.3% (samples from Jiroft). The content of linoleic acid, also, ranged between 33.7% and 36.3% and 32.3% and 36% for sour lemon and orange, respectively. The rest of the properties of the seeds are listed in Table 2. As indicated, average amount of iodine, peroxide, acid, and saponification in sour lemon is 98.62%, 0.62%, 0.54%, and 190.43%; these figures for orange are 101.94%, 0.65%, 0.57%, and 188.2%, respectively.

## 4. Discussion

The considerable content of unsaturated fatty acid in oil of the seeds hints at the great nutritional value of the oil so that linoleic acid is one of the 3 essential FAs. In comparison, the oil of sour lemon seeds is the best option regarding unsaturated fatty acid (nutritional value). Regarding the composition of the oil from different species and varieties, this study found the samples less different comparing with the results of other similar studies. Researches that have been conducted on properties of citrus seed oils are as follows. Anwar et al. found that linoleic acid is the main acid in citrus seed oil (36.1–39.8%) and the other key fatty acids were palmitic acid (25.8–32.2%), oleic acid (21.9–24.1%), linolenic acid (3.4–4.4%), and stearic acid (2.8–4.4%) [7]. In a study by Matthaus and Ozcan, the oil content of seeds was reported somewhat between 32.1 g/100 g and 58.8 g/100 g. In descending order, the main fatty acids in the oil samples were oleic (12.8–70.1%), linoleic (19.5–58.8%), and palmitic (5.1–28.3%). Furthermore, content of stearic, vaccenic, linolenic, and arachidic acids was negligible [12]. Mahmud et al. reported good quality of unsaturated acids found in citrus fruit oil (49.92%). Such high content of unsaturated acid is expected in a quality edible oil [13]. In another study, Saloua et al. indicated that linoleic (76.19%), oleic (13.87%), stearic (6.76%), and palmitic acids (2.40%) are the key fatty acids found in crude oil [14]. Waheed et al. showed that, in sum, content of lipids C18:3 was 4.66% in* C. aurantium* and 3.58% in *C. paradisi* [15]. Our results are similar in fatty acid composition when compared to the values in the literature. As the results discussed herein showed, orange and sour lemon seeds oil is a rich source of unsaturated fatty acids so that citrus seed oil can be of the best oils for man.

## 5. Conclusion

Nutritionally speaking, citrus seed oil is a notable choice so that average unsaturated fatty acids found in sour lemon and orange varieties are 66.46% and 68.83%, respectively. Our results indicated that the oil extracted from citrus seeds grown in Iran is a rich source of essential fatty acids and is of high potential for being used for nutritional and industrial usages. As one of the biggest producers of sour lemon and orange, the seeds of the fruits in Iran, which are currently disposed of, can be a reliable source of edible oil.

## Figures and Tables

**Table 1:** Fatty acid compositions of the oils extracted from different citrus seed species (%).

	Palmitic	Palmitoleic	Stearic	Oleic	Linoleic	Linolenic	Other fatty acids	Oil content
Qaleh Ganj								
Lemon	29.4	0.7	4.7	26.4	34.1	6.2	0.5	41.5
Citrus	27.6	0.6	6.5	27.1	34	3.2	1	34.1

Jiroft								
Lemon	27.8	0.9	4.1	24.8	35.7	7	0.6	41.9
Citrus	27.3	0.4	4.8	29.3	36.3	3.3	0.9	37.2

Anbarabad								
Lemon	23.5	0.6	4.2	28.5	33.7	7.8	1.4	40.3
Citrus	26.5	0.6	6.5	28.6	32.2	4.1	1.5	33.4

**Table 2:** Physicochemical characteristics of oils from citrus seed species.

	Iv^1^	SN^2^	PV^3^	AV^4^
Qaleh Ganj				
Lemon	97.32	192.4	0.54	0.48
Citrus	99.65	189.2	0.63	0.52

Jiroft				
Lemon	98.17	191.3	0.55	0.44
Citrus	102.41	186.5	0.69	0.61

Anbarabad				
Lemon	100.38	187.6	0.77	0.72
Citrus	103.77	188.9	0.64	0.58

^1^Iodine value (I2 g per 100 g of oil).

^2^Saponification number (mg KOH per g of oil).

^3^Peroxide value (mequiv O_2_ per kg of oil).

^4^Acid value (mg KOH per g of oil).

## References

[1] Khan IA (2007). *Citrus Genetics, Breeding and Biotechnology*.

[2] El-Adawy TA, Rahma EH, El-Bedawy AA, Gafar AM (1999). Properties of some citrus seeds. Part 3. Evaluation as a new source of protein and oil. *Food/Nahrung*.

[3] Vand SH, Abdullah TL (2012). Identification and introduction of thornless lime (Citrus aurantifolia) in Hormozgan, Iran. *Indian Journal of Science and Technology*.

[4] Saïdani M, Dhifi W, Marzouk B (2004). Lipid evaluation of some Tunisian Citrus seeds. *Journal of Food Lipids*.

[5] Reda SY, Leal ES, Batista EAC (2005). Characterization of rangpur lime (*Citrus limonia* Osbeck) and “sicilian” lemon (*Citrus limon*) seed oils, an agroindustrial waste. *Ciência e Tecnologia de Alimentos*.

[6] Habib MA, Hammam MA, Sakr AA, Ashoush YA (1986). Chemical evaluation of egyptian citrus seeds as potential sources of vegetable oils. *Journal of the American Oil Chemists’ Society*.

[7] Anwar F, Naseer R, Bhanger MI, Ashraf S, Talpur FN, Aladedunye FA (2008). Physico-chemical characteristics of citrus seeds and seed oils from Pakistan. *Journal of the American Oil Chemists' Society*.

[8] Zhang QA, Zhang Z, Yue X, Fan X, Li T, Chen S (2009). Response surface optimization of ultrasound-assisted oil extraction from autoclaved almond powder. *Food Chemistry*.

[9] AOCS (2009). *Official Methods and Recommended Practices of the American Oil Chemists’ Society*.

[10] Moayedi A, Rezaei K, Moini S, Keshavarz B (2011). Chemical compositions of oils from several wild almond species. *Journal of the American Oil Chemists' Society*.

[11] AOAC (2005). *Association of Official Analytical Chemists*.

[12] Matthaus B, Ozcan MM (2012). Chemical evaluation of citrus seeds, an agro-industrial waste, as a new potential source of vegetable oils. *Grasas y Aceites*.

[13] Mahmud S, Akhtar H, Saleem M, Khanum R (2009). Lipid classes of in vitro cultivar of Pakistani citrus. *Journal of Saudi Chemical Society*.

[14] Saloua F, Eddine NI, Hedi Z (2009). Chemical composition and profile characteristics of Osage orange *Maclura pomifera* (Rafin.) Schneider seed and seed oil. *Industrial Crops and Products*.

[15] Waheed A, Mahmud S, Saleem M, Ahmad T (2009). Fatty acid composition of neutral lipid: classes of Citrus seed oil. *Journal of Saudi Chemical Society*.

